# Revisiting the Optical Response of Two-Dimensional Perovskites: Beyond Excitons

**DOI:** 10.1021/acs.nanolett.6c01074

**Published:** 2026-06-19

**Authors:** Alessandro Surrente, Mateusz Dyksik, Paulina Plochocka, Michał Baranowski

**Affiliations:** † Department of Experimental Physics, Wrocław University of Science and Technology, Wybrzeże Wyspiańskiego 27, Wrocław 50-370, Poland; ‡ Laboratoire National des Champs Magnétiques Intenses, EMFL, CNRS UPR 3228, Université Grenoble Alpes, Université Toulouse, Université Toulouse 3, INSA-T, Grenoble and Toulouse 38042, France

**Keywords:** 2D perovskites, exciton and photoluminescence, dielectric function, semiconductors, Reststrahlen effect

## Abstract

Two-dimensional (2D) Ruddlesden–Popper metal halide perovskites exhibit an unusually rich optical response, characterized by multiple sidebands, broad quasi-plateaus, and pronounced thickness-dependent spectral features. In this Review, we reassess their optical properties by examining the interplay between electronic structure, exciton fine structure, exciton–phonon coupling, and photonic effects. We show that the exceptionally large excitonic oscillator strength and high refractive index naturally give rise to excitonic stop bands and interference effects that can dominate reflection, transmission, and absorption spectra, even in nominally free-standing crystals. We further discuss the complexity of the photoluminescence response, including evidence for exciton–polaron formation, self-trapping, and defect-assisted recombination. By placing electronic, vibrational, and photonic effects on equal footing, this Review provides a unified framework for interpreting optical spectra in 2D perovskites.

## Graphical abstract

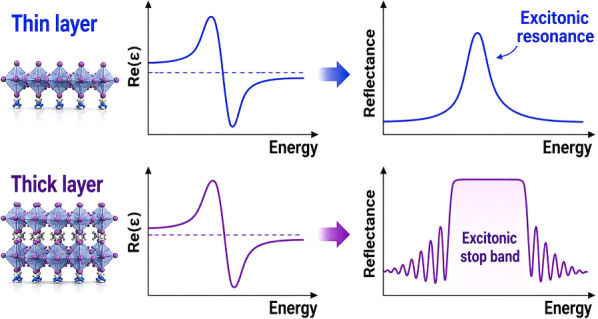

Two-dimensional (2D) organic inorganic metal halide Ruddlesden–Popper (RP) perovskites^1^ form a subclass of van der Waals semiconductors,^2^ alongside materials such as transition-metal dichalcogenides (TMDs),^3,4^ black phosphorus,^5^ and emerging layered magnetic semiconductors.^6^

They are described by the general chemical formula^1^ *R*_2_ *A*_*n*–1_ *B*_*n*_ *X*_3*n*+1_, where *R* denotes a bulky organic spacer cation, *A* is a small monovalent cation (e.g., MA or FA), *B* is a divalent metal (typically Pb or Sn), and *X* is a halide. The structure consists of atomically thin inorganic metal-halide slabs composed of corner-sharing *BX*_6_ octahedra, separated by organic spacer layers that are held together by van der Waals interactions, forming a natural multiple-quantum-well architecture.^1,7^ The parameter *n* defines the thickness of the inorganic slab and corresponds to the number of octahedral layers confined between adjacent organic barriers. Increasing *n* therefore increases the quantum-well thickness and drives a gradual evolution of the electronic and optical properties toward the three-dimensional perovskite limit. Among the most extensively studied representatives are phenethylammonium (PEA)-based and butylammonium (BA)-based lead iodides; however, the range of possible organic spacer cations extends far beyond these two examples.^1,8^ This structural diversity provides an exceptionally versatile platform for tailoring the optoelectronic properties of these materials through structural templating.^8–10^

A hallmark of 2D layered metal halide perovskites is their robust excitonic optical response driven by the interplay of quantum and dielectric confinement,^16–19^ which significantly enhances Coulomb interactions and leads to exceptionally large exciton binding energies, reaching several hundreds of meV for the thinnest quantum wells (*n* = 1).^17,20^ As a result, excitonic resonances characterized by exceptionally large oscillator strength remain stable even at room temperature and shape the particularly complex optical response on equal footing with strong exciton–phonon coupling^14,18,21^ and photonic effect.^15,22^

Indeed the experimentally reported absorption or reflection spectra of 2D perovskites are remarkably rich and complex,^7,10–12,14,16,20,23–33^ in clear contrast to other archetypal 2D layered semiconductors such as, for example, TMDs.^4^ For instance, the low-temperature absorption and reflectivity spectra of PEA_2_MA_*n*–1_Pb_*n*_I_3*n*+1_ shown in Figure 1(a,b) are characterized by substantial broadening and the dominant excitonic feature is commonly, followed by high energy sidebands separated by approximately 35–40 meV (see, e.g., the spacing Δ in Figure 1(a)). Such behavior appears to be a general characteristic of layered perovskites independently on wide variety of inorganic frameworks and organic spacer cations.^10,12,14,20,23,24,27,29,32^

**Figure 1:**
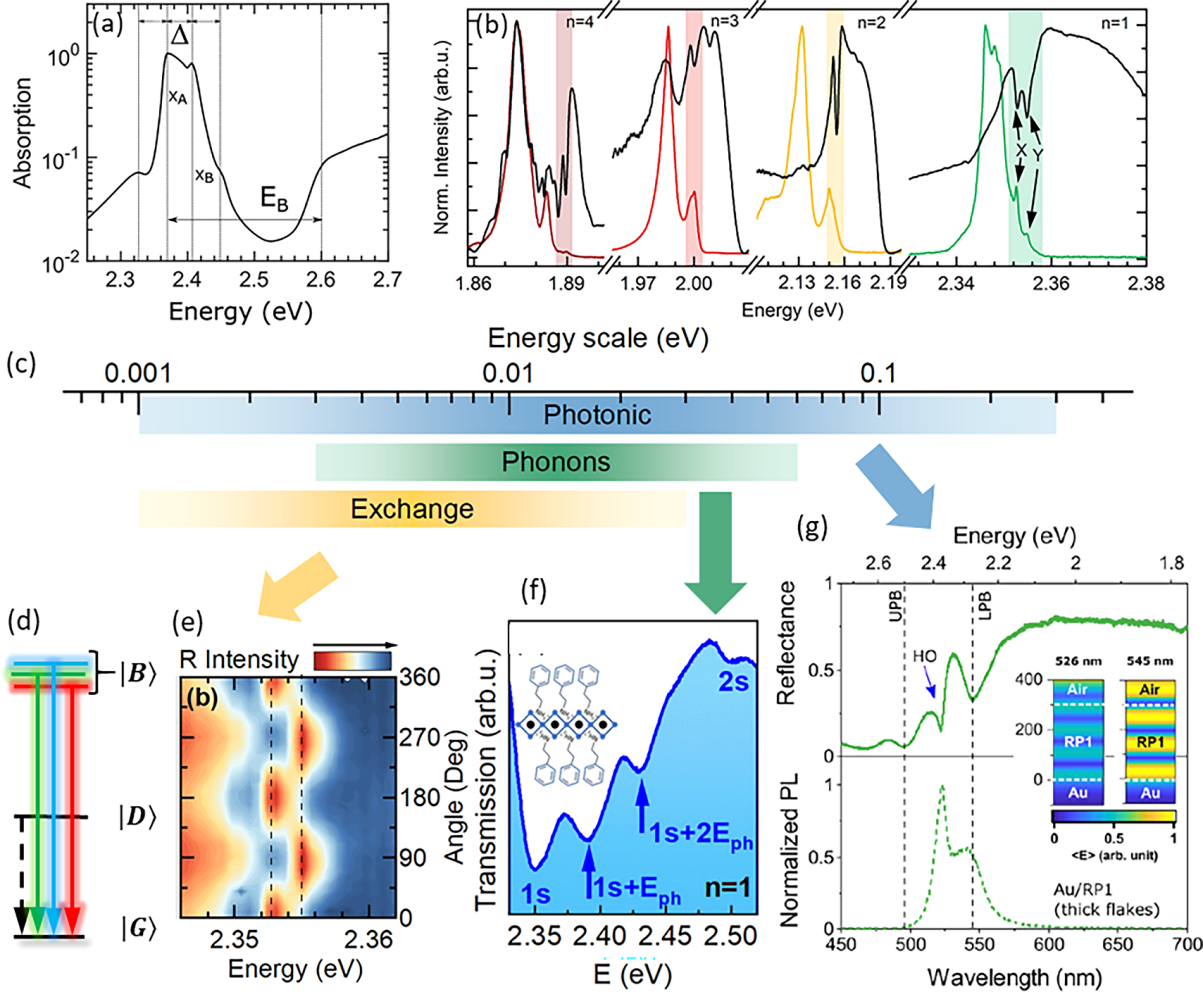
(a) Absorption spectrum of (PEA)_2_PbI_4_ measured at *T* = 5 K. The spacing Δ ≈ 35 meV indicates the characteristic separation of spectral features commonly observed in absorption, reflectance, and transmission spectra. [Reproduced from ref ^11^. Copyright 2020, American Chemical Society.] (b) Photoluminescence (colored lines) and reflectance (black lines) spectra of PEA_2_MA_*n*–1_Pb_*n*_I_3*n*+1_. Broad multipeak structures are evident in the reflectance spectra for *n* = 2, 3, 4. [Reproduced from ref 12. Copyright 2024, American Chemical Society.] (c) Illustration of characteristic energy scales associated with exchange interaction, exciton–phonon coupling, and photonic effects. (d) Schematic representation of the bright exciton triplet and dark singlet states expected for 2D perovskites. (e) Evolution of the reflectance spectrum as a function of the angle between the incident light polarization and the in-plane crystal axis. [Reproduced from ref 13. Copyright 2022, American Chemical Society.] (f) Transmission spectra of (PEA)_2_PbI_4_ at *T* = 4 K showing characteristic equally spaced minima. [Reproduced from ref 14. Copyright 2020, American Chemical Society.] (g) Reflectance and photoluminescence spectra of an ∼300-nm-thick (BA)_2_PbI_4_ flake deposited on a gold substrate. The formation of lower and upper polariton branches is visible in both reflectance and photoluminescence. [Reproduced from ref 15. Copyright 2021, American Chemical Society.]

In general, the optical response of a semiconductor near its fundamental bandgap is shaped by three principal factors: (*i*) the underlying electronic structure, (*ii*) the coupling between electronic states and lattice vibrations, and (*iii*) the photonic environment in which the dipole is embedded. While the first two aspects are routinely invoked to rationalize the intrinsic optical response of 2D perovskites, the role of photonic effects is often underestimated. Although 2D perovskites are widely recognized as promising platforms for exploring strong light–matter interactions when embedded in optical cavities,^15,34–39^ the potential complexity of their optical response arising from the exceptionally large excitonic oscillator strength^22,34,40^ and relatively high refractive index^40^ is frequently overlooked when considering intrinsic material response (free-standing crystal). Importantly, because 2D perovskites exhibit negligible electronic coupling between their inorganic layers^17–19^ (in contrast with other van der Waals materials^3,5,6^ ), they behave as an independent multiple quantum wells structure. Therefore, strongly bound quasi-2D excitons can be investigated in both ultrathin exfoliated flakes^41–43^ and bulk-like crystals,^13,20,24,29^ where the physical thickness of the structure itself exerts a pronounced influence on the optical response.

Crucially, as summarized in Figure 1(c), the characteristic energy scales associated with these different contributions partially overlap in 2D perovskites. This overlap likely constitutes one of the primary sources of the ongoing debate and controversy surrounding the interpretation of optical spectra and, consequently, the microscopic origin of the observed transitions.^7,11,14,18,21,29^ As discussed in this Review, the attribution of specific spectral features to individual electronic, vibronic, or photonic contributions must therefore be approached with particular caution. In the following, we systematically examine the role of each of these factors and analyze how their interplay shapes the observed optical response.

Theoretical predictions and symmetry analyses indicate that the band structure near the bandgap in both three-dimensional (3D) and 2D metal halide perovskites is similar.^25,26,44–46^ The edge of the conduction band is primarily derived from lead *p*-type orbitals, whereas the top of valence band has *s*-type character (with admixture of the halide *p*-type orbital).^26,45,47,48^ The degeneracy of the conduction-band states, characterized by different angular momentum projections, is lifted by spin–orbit coupling and crystal-field interactions, particularly in lower-symmetry phases such as tetragonal or triclinic structures.^44,48^ As a consequence, the optical spectra near the band edge are dominated by the interband excitonic transitions involving holes from the top of the valence band (*S* = 1/2, *S*_*z*_ = ±1/2) and electrons from the lowest spin–orbit-split conduction band (*J* = 1/2, *J*_*z*_ = ±1/2).^26,44–46^ Higher conduction bands are typically separated from the lowest one by hundreds of meV or more,^17^ and therefore do not significantly influence the optical response.

The four possible band-edge excitonic states, corresponding to different combinations of electron and hole angular momenta, form a bright triplet and a dark singlet manifold,^25,26,44–46,49,50^ as schematically illustrated in Figure 1(d). Depending on the crystal symmetry, these states may exhibit different degrees of degeneracy.^44^ For instance, in the archetypal (PEA)_2_PbI_4_, the degeneracy of the bright excitonic states is fully lifted,^13,25,51^ and the optical response is governed by three linearly and orthogonally polarized excitonic transitions. This picture of exciton fine structure in 2D perovskites has been corroborated by numerous experimental studies.^12,13,25,31,33,42,49,51^

In general, for the thinnest (*n* = 1) 2D perovskites, the optically inactive dark singlet lies few tens of meV below the bright states,^25,33,49,52,53^ whereas the fine-structure splitting within the bright triplet usually does not exceed a few meV.^12,13,31,42,51^ Consequently, it can be well resolved only at low temperatures in high quality (single) crystals.^12,13,31,42,51^ As a representative example, the fine-structure splitting of the in-plane bright exciton states of (PEA)_2_PbI_4_ is shown in Figure 1(e). On a more extended energy scale (or lower quality polycrystalline thin films), the bright states can often be treated as effectively degenerate and approximated by a single excitonic transition. Importantly, the magnitude of the fine-structure splitting is far smaller than the ∼30–40 meV separation commonly observed between sidebands in optical spectra (see Figure 1(a)). Therefore, the exciton fine structure of bright states alone cannot account for these higher-energy features, as their energy scale substantially exceeds that expected from exchange interaction.

Because of the considerations discussed above, strong exciton–phonon coupling is frequently invoked to rationalize the broad and structured optical response of 2D perovskites. Indeed, there is broad agreement that their rich vibrational spectrum^21,54,55^ and the pronounced coupling between excitons and optical phonons play a crucial role in shaping the optical response, both in 3D metal halide perovskites^56,57^ and in reduced-dimensional systems.^7,14,18,30,58,59^ These interactions form the basis for several competing interpretations proposed in the literature. One widely discussed explanation attributes the nearly equally spaced sidebands, or minima in the transmission spectra (see Figure 1(f)), to phonon replicas of the primary excitonic transition.^7,10,14,32,58,60^ Within this framework, additional spectral features arise from excitonic recombination or absorption processes accompanied by the emission (particularly at low temperatures) of one or more optical phonons, producing a vibronic progression in the optical spectrum. The resulting structure reflects the quantized coupling between excitonic states and lattice vibrations. Interestingly, the observed energy spacing of approximately 30–40 meV suggests the involvement of relatively high-energy phonon modes. This is somewhat surprising given the generally soft lattice of metal halide perovskites, where most optical phonons typically lie in the few-meV range,^21,54^ and Raman spectra are dominated by low-energy modes. This apparent discrepancy has been rationalized by the coupling between excitons localized in the inorganic framework and higher-energy vibrational modes of the organic spacer cations.^7^

Alternatively, several studies attribute the observed spectral spacing to the polaronic nature of photoexcited carriers in 2D perovskites.^11,21,24^ In this picture, electronic excitations are intrinsically coupled to lattice distortions, leading to the formation of exciton-polaron quasiparticles. The multiple spectral features, such as the X_*A*_ and X_*B*_ resonances indicated in Figure 1(a), are then interpreted as distinct exciton-polaron states, whose Coulomb interactions are renormalized by local lattice deformation.^11,21,24^ In this scenario, lattice dressing becomes an integral component of the excitonic eigenstates, giving rise to a family of coexisting excitonic species with distinct binding energies and optical signatures, thereby challenging the interpretation based solely on vibronic replicas of a single excitonic transition.

Recent Raman studies provide additional insight into this issue, showing that the phonon scattering spectrum changes dramatically under electronic excitation.^55^ This behavior has been interpreted as evidence of polaron formation. Notably, the enhanced Raman response appears in an energy range consistent with the spacing of spectral features observed in optical absorption and reflection measurements. This observation suggests that multiple low-energy phonon processes may collectively contribute to the observed spectral structure, potentially bridging the phonon-replica and exciton-polaron interpretations.

Importantly, regardless of the microscopic origin assigned to the multiple excitonic resonances, rationalizing the overall shape of reflection or transmission, or absorption spectra in 2D perovskites remains challenging. In particular, the frequently observed broad, relatively flat plateau around the excitonic transition energy is difficult to explain solely by invoking multiple excitonic transitions. This suggests that an essential ingredient may still be missing in many interpretations. As discussed below, photonic effects can play a crucial role not only in the strong-coupling regime, where exciton-polaritons dominate the optical response^15,38^ (see Figure 1(g)), but also in nominally uncoupled structures. In particular, the exceptionally large excitonic oscillator strength, combined with the relatively high refractive index of 2D perovskites, can lead to the emergence of a excitonic stop band (Reststrahlen effect) that naturally accounts for several observed spectral features. In the following, we therefore examine step by step how these photonic effects contribute to the complexity of the optical response and why they must be considered alongside microscopic excitonic models.

To model the optical response, we employ a semiclassical description of exciton–photon interaction based on the susceptibility formalism for the dielectric function.^15,61^ Close to the excitonic resonance, the dielectric response is described using a Lorentz oscillator model, while contributions from higher-energy electronic transitions are incorporated through a frequency-independent background permittivity ε_*∞*_:

1 $$\varepsilon \left(E\right)=\varepsilon_{\infty }+\frac{f_{X}}{E_{0}^{2}-E_{X}^{2}-i\Gamma E}$$

Here, *E*_*X*_ denotes the excitonic transition energy, *f*_*X*_ is the corresponding oscillator strength, and Γ represents the phenomenological broadening (damping) parameter. The parameters used to model the dielectric function are summarized in Table 1. Those of nonphenomenological nature have been extracted from prior experimental work.^34^ The resulting real and imaginary parts of the dielectric function are shown in Figure 2(a,b).

**Figure 2:**
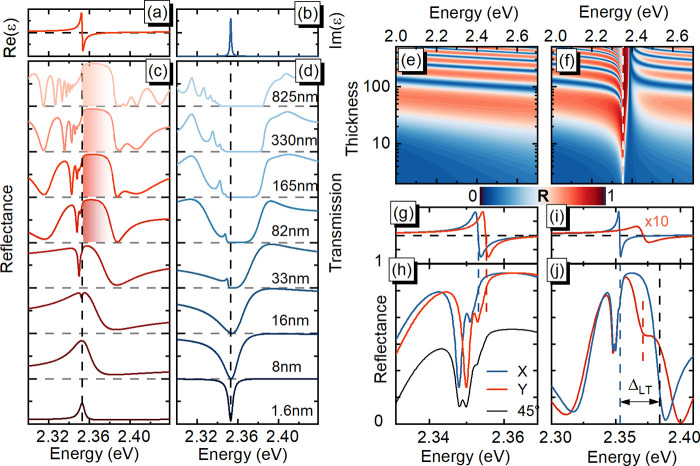
(a) Real and (b) imaginary parts of the dielectric function calculated using eq 1. (c) Corresponding reflection and (d) transmission spectra for varying thicknesses of the perovskite layer, computed using the parameters listed in Table 1. The dashed lines indicate the energy of the excitonic transition. The Reststrahlen band is schematically indicated by the shaded region in panel (c) for thicker layers. (e) False-color map of the reflection spectrum as a function of layer thickness, calculated assuming a constant background dielectric screening ε_∞_ = 5.76, illustrating cavity-mode formation. (f) Modification of the reflection spectrum when the excitonic resonance is included in the dielectric response. (g) Two components of the dielectric function along the *X* and *Y* crystallographic directions. (h) Corresponding reflectance spectrum for an ∼82-nm-thick layer assuming light polarized along the *X* (blue) and *Y* (red) axes. The black curve (shifted downward for clarity) corresponds to light polarized at 45° with respect to the *X* and *Y* axes. (i) Two components of the dielectric function corresponding to excitonic transitions at *E*_*X*_ and $E_{X}^{\prime }$ , as specified in Table 1. (j) Reflection spectrum of an ∼82-nm-thick layer calculated assuming either a single oscillator (*E*_*X*_, blue) or two oscillators (*E*_*X*_ and $E_{X}^{\prime }$ , red). The dashed lines indicate the longitudinal–transverse splitting associated with the *E*_*X*_ transition.

**Table 1:** Dielectric and Excitonic Parameters Used in the Optical Model^a^

*ε*_*∞*_	*E*_*X*_ (eV)	*f*_*X*_ (eV^2^)	Γ (eV)	$E_{X}^{\prime }$ (eV)	$f_{X}^{\prime }$ (eV^2^)	Γ′ (eV)
5.76^b^	2.353	0.85^b^	0.0015	2.371	0.2	0.020

a The parameters $E_{X}^{\prime }$ , $f_{X}^{\prime }$ , and Γ′ correspond to the additional oscillator introduced to model the impact of extra transitions related to exciton-phonon coupling, as presented in Figure 2(i,j).

b Data taken from ref 34.

Solving Maxwell’s equations within the transfer-matrix formalism^62,63^ for the energy-dependent dielectric function, we calculate the reflectivity and transmission spectra for perovskite layers of varying thickness, shown in Figure 2(c,d). The reflectivity simulations correspond to perovskite layers deposited on a 90 nm SiO_2_ layer on a Si substrate, whereas the transmission calculations assume a free-standing perovskite layer. Normal incidence of the incoming light is assumed throughout.

The simulations presented in Figure 2(c,d) show that only in the monolayer (approximately 1.6 nm for (PEA)_2_PbI_4_ ^64^) and few-layer limit do the reflectivity and transmission spectra closely resemble the dispersive and absorptive line shapes associated with the real and imaginary parts of the dielectric function, respectively. The simulated response for very thin layers resembles experimental spectra reported for exfoliated flakes.^41^ As the layer thickness increases, the excitonic resonance in reflectivity broadens, and the spectral line shape becomes progressively more complex, consistent with observations for thicker films or bulk crystals,^10,11,16,20,21,24–26,33^ also shown in Figure 1. Characteristic plateaus, where reflectivity approaches unity and transmission tends toward zero, develop with increasing thickness. In addition, interference-induced oscillatory features emerge on the low-energy side of the excitonic transition for sufficiently thick layers.

The emerging plateau corresponds to a photonic stop band, or Reststrahlen band,^65–67^ which occurs when the real part of the dielectric function becomes negative over a certain energy range. In this regime, the refractive index becomes predominantly imaginary and the reflectance approaches unity, producing the characteristic spectral plateau. Notably, the large oscillator strength, approaching ∼1 eV^2^ (a characteristic feature of *n* = 1 2D perovskites^15,22,34,35,40^ ), causes this plateau to extend over a few tens of meV. This effect, well-known in bulk polar crystals^65^ and recently highlighted in van der Waals materials such as hBN,^66,67^ CrSBr,^68^ or ReS_2_,^69^ can likewise be expected for the parameter range characteristic of 2D perovskites. Simultaneously, the rapid increase of the refractive index as the photon energy approaches the excitonic resonance induces a substantial phase shift (∼*nd*) upon each pass through the layer, leading to interference fringes on the low-energy side of the excitonic transition.

Furthermore, the optical spectrum of thicker 2D perovskite layers can be significantly influenced by thin-film interference effects. Owing to their relatively high refractive index (*n* ≈ 2.4),^34,40^ these layers can support Fabry-Pérot-like photonic modes whose energies depend sensitively on layer thickness, as illustrated in Figure 2(e). Although a free-standing perovskite layer-i.e., in the absence of external mirrors or a deliberately engineered cavity–does not typically reach the strong exciton–photon coupling regime,^15^ noticeable modifications of the optical response can still occur when the photonic mode energy approaches the excitonic transition energy (see Figure 2(f)). In this situation, the reflectivity spectrum, including the position, width, and depth of spectral features, exhibits a pronounced thickness dependence, reflecting the detuning between the photonic mode and the excitonic resonance.

Importantly, these simulations, even though they assume only a single excitonic resonance, capture the main qualitative features of the optical response of 2D perovskites in both the ultrathin^41^ and bulk-like limits (see Figure 1). In particular, they reproduce the broad quasi-plateau behavior of the reflectance or transmission around the excitonic transition energy (as seen in Figure 1(a,b)), as well as the characteristic dips or oscillatory features preceding the onset of the stop band. These results demonstrate that even a single excitonic transition with sufficiently large oscillator strength can generate a remarkably complex optical response through purely photonic effects, strongly dependent on the thickness of the structure.

When taking into account more than one oscillator, whether arising from the exciton fine structure or exciton–phonon coupling, it further complicates the picture. For instance, exciton fine-structure splitting introduces multiple closely spaced optical transitions with different dipole orientations.^13,22,45,49,51^ This leads to anisotropic dielectric properties that differ along two orthogonal in-plane crystallographic directions, as illustrated in Figure 2(g). Such anisotropy can manifest in reflection spectra as a splitting or modification of the interference fringes appearing near the onset of the stop band, as shown in Figure 2(h) and reported in numerous experimental studies^12,13,31,51,60^ (see also Figure 1(d) for the *n* = 1 case). When polarized light is used to probe the optical response, the spectra shift depending on the relative orientation between the incident polarization and the crystal axes (see Figure 1(e)).

Moreover, while the simulations presented here assume normal incidence, in typical experiments, the detected light is collected over a finite solid angle determined by the numerical aperture of the objective or the lens. Under these conditions, the optical spectra may be further modified, particularly because one of the bright exciton states often possesses an out-of-plane dipole moment. This results in different responses for TE- and TM-polarized components at oblique incidence, adding another layer of complexity to the measured spectra.^22^

It is also important to note that a stop band arising from a single excitonic resonance is generally insufficient to account for the additional high-energy sidebands frequently observed above the onset of the main excitonic feature,^10,11,31,60^ as well as other small features appearing on top of the stop band.^12,24,29,33^ The width of the excitonic stop band is primarily determined by the oscillator strength of the transition^67^ and, in the limit of negligible damping, is approximately given by the exciton longitudinal–transverse splitting Δ_LT_ (see Figure 2(b)).^67^ This is formally analogous to the longitudinal-transverse splitting of optical phonons that defines the Reststrahlen band in the infrared spectral range.^66^ For typical parameters (*f*_*X*_ ≈ 0.85–1 eV^2^ and ε_*∞*_ ≃ 5.8, *E*_*X*_ = 2.353),^34^ the expected stop-bandwidth (assuming negligible Γ):^67,68^

2 $$\Delta_{LT}=\frac{f_{X}}{2\varepsilon_{\infty }E_{X}}$$

does not exceed roughly 30–35 meV. However, experimental spectra of *n* = 1 2D perovskites often exhibit sidebands extending beyond this range^10,11,60^ (see, for example, Figure 1(a)), suggesting that the principal excitonic transition is accompanied by additional higher-energy resonances. Indeed, as illustrated in Figure 2(i,j), incorporating an additional resonance separated by ∼20 eV into the dielectric response produces a characteristic high-energy sideband adjacent to the main stop band. While the present analysis does not resolve the microscopic origin of these additional oscillators, it demonstrates that reliable identification of their energies and physical nature requires careful consideration of the overall optical response, including photonic effects.

We emphasize that the parameters used in the presented analysis correspond to experiments performed on high-quality single crystals at low temperatures. In this regime, the optical response is well described by narrow excitonic resonances, allowing photonic effects to be clearly resolved. However, their importance also extends to conditions where both homogeneous and inhomogeneous broadening affect the optical response.

We first consider the effect of increased homogeneous broadening, described by the damping parameter Γ (see Figure 3(a)), as expected at elevated temperatures. As shown in Figure 3(a), increasing Γ reduces the amplitude of the dielectric response around the excitonic transition. As a consequence, the reflectance decreases (due to a reduced refractive index), and the stop band becomes progressively less pronounced (see Figure 3(b)). In particular, both the low- and high-energy edges of the stop band become smoother, and for sufficiently large Γ the dielectric function no longer reaches negative values. In this regime, the reflectance recovers a more dispersive line shape, resembling the underlying dielectric response. Importantly, even for relatively large Γ, the spectral width of the excitonic feature in reflectance remains primarily governed by the longitudinal–transverse splitting Δ_LT_, rather than by Γ.

**Figure 3:**
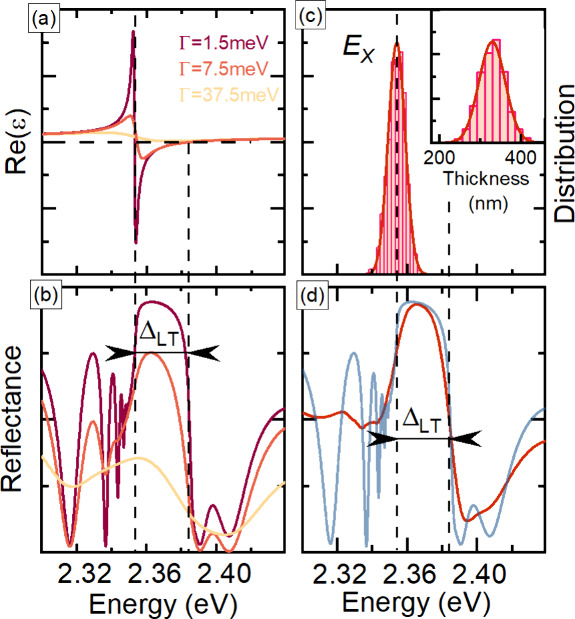
(a) Real part of the dielectric function calculated using eq 1 for different values of the damping parameter Γ. (b) Corresponding reflectance spectra for a perovskite layer with a thickness of 330 nm. The remaining parameters are listed in Table 1. The dashed lines indicate the excitonic transition energy and the longitudinal–transverse splitting Δ_LT_. (c) Histogram of excitonic transition energies *E*_*X*_ used to simulate the reflectance spectrum of a polycrystalline film. The inset shows the corresponding distribution of grain thicknesses. (d) Reflectance spectrum: the red curve represents the response averaged over 1000 grains with randomly distributed thickness and exciton energy. The blue curve corresponds to the reflectance of a single oscillator with mean values of the exciton energy and layer thickness.

We further consider the case of polycrystalline films, where the measured reflectance represents an average over regions with varying thickness and local fluctuations of the excitonic transition energy, two key sources of inhomogeneous broadening. We model this situation by assuming a normal distribution of thickness (mean = 330 nm, σ = 20 nm) and exciton energy (mean = 2.354 eV, σ = 5 meV), see Figure 3(c), while keeping a small intrinsic line width (Γ = 1.5 meV) for each grain, corresponding to low-temperature conditions. As shown in Figure 3(d), averaging over many grains primarily suppresses the interference fringes preceding the onset of the stop band (compare red and blue curves), consistent with their strong sensitivity to thickness variations. In contrast, the main stop-band feature remains clearly visible, although its edges become less sharp due to the distribution of excitonic transition energies. Notably, the overall spectral width of the reflectance feature remains governed by the oscillator strength, i.e., the longitudinal–transverse splitting Δ_LT_.

These results indicate that while increased broadening and disorder reduce fine spectral details, the formation of the stop band itself remains a robust feature of the optical response. Consequently, photonic effects associated with large excitonic oscillator strength can remain relevant even in disordered systems such as polycrystalline films or at room temperature, although their spectral signatures become progressively less pronounced.

The considerations presented above highlight that photonic effects can substantially shape the optical response of 2D perovskites. As a result, particular care is required when assigning spectral features from both single- and polycrystalline structures. The thickness sensitivity, inherent to photonic effects in excitonic media, is a defining characteristic of these materials and can significantly complicate the interpretation of reflection, transmission, or absorption spectra. We note that, in the case of polycrystalline films, recent advances in nano-optical techniques such as cathodoluminescence^70,71^ and near-field nanospectroscopy^72,73^ can help mitigate the challenges arising from the interplay between photonic and disorder effects (for instance, thickness variation or grain boundaries) by enabling a direct correlation between local structure and spectral features.

Consequently, resolving the full set of excitonic resonances, including exciton-polaron states or phonon replicas, would greatly benefit from studies on high-quality exfoliated single-crystal flakes with minimal thickness. We note that investigations of ultrathin exfoliated 2D perovskites can be challenging due to their sensitivity to ambient conditions and illumination.^64^ Nevertheless, a growing number of recent studies demonstrate that careful exfoliation in a glovebox, combined with hBN encapsulation, enables stable optical investigations of such systems.^42,74,75^ This makes it feasible to probe the intrinsic excitonic response in the ultrathin limit, where thickness-dependent photonic effects are minimized. For instance, these challenges are generally less pronounced in TMDs, where most studies focus on the monolayer or few-layer limit.^4,76^ In this condition, the reduced thickness simultaneously minimizes thickness-dependent photonic contributions and simplifies spectral interpretation.

We emphasize that the presented discussion is not limited to lead–iodide systems but extends to a broader class of 2D metal halide perovskites, including bromide-, chloride-, and Sn-based analogues^29^ or Dion-Jacobson phase.^40^ Owing to their similar layered structure and the resulting quantum and dielectric confinement, these materials also exhibit strongly bound excitons^33^ with large oscillator strengths,^40^ which are the key ingredients for the emergence of photonic stop-band effects. While differences in halide composition primarily modify the relevant energy scales such as exciton binding energy^10,20,33,49^ or background dielectric screening,^40^ the underlying photonic mechanisms remain unchanged. Generally, this picture is applicable to any strongly excitonic material with sufficiently large oscillator strength.^15,67,68^

Having discussed the complexity of the reflection and transmission spectra, we now turn to the emission spectrum of 2D perovskites, which is also highly complex. The photoluminescence (PL) of these materials spans a remarkably broad energy range, as shown in Figure 4(a).^77^ It typically consists of a slightly red-shifted emission close to the excitonic resonance observed in absorption,^13,77^ and by broad bands that can be shifted by several hundreds of meV toward lower energies.^77–86^ Such a wide spectral distribution of emission features indicates that radiative recombination in 2D perovskites cannot be described solely in terms of free or weakly perturbed excitons.

**Figure 4:**
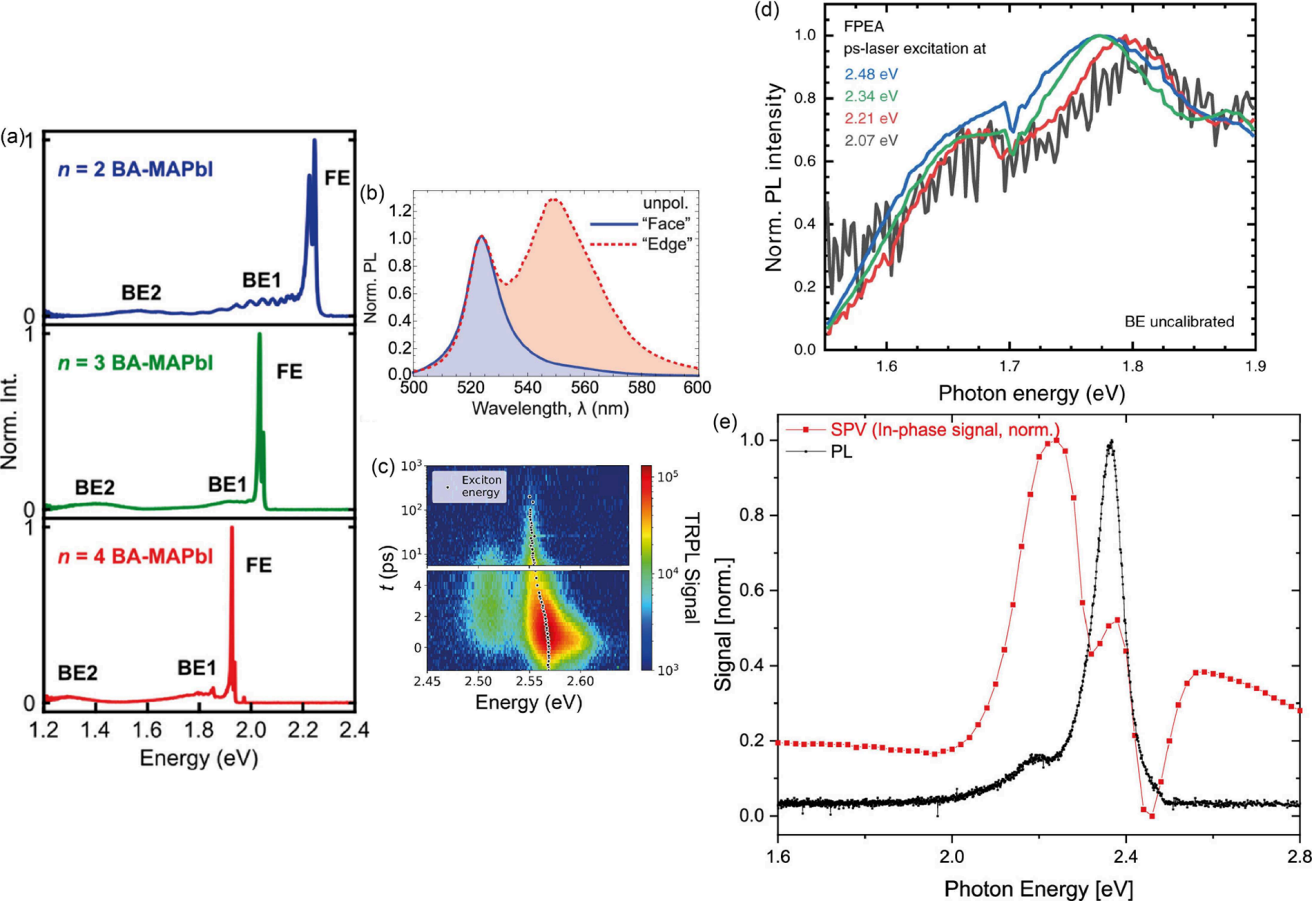
(a) PL spectrum of BA_2_MA_*n*–1_Pb_*n*_I_3*n*+1_, *n* = 2 (top, blue curve), *n* = 3 (center, green curve) and *n* = 4 (bottom, red curve). FE denotes the emission from the recombination of free excitons. Two broad sub-band emission peaks are denoted by BE1 and BE2, respectively. [Reproduced from ref 77. Copyright 2020, American Chemical Society.] (b) PL spectrum of PEA_2_PbI_4_ measured in normal incidence configuration (parallel to the *c* axis of the crystal, blue) and measured from the edge of the crystal (perpendicular to the *c* axis, red). [Reproduced from ref 87. Copyright 2020, American Chemical Society.] (c) Low temperature spectrally resolved, time-resolved PL spectrum of (2-BrPEA)_2_PbI_4_. The black dot keep track of the energy position of the exciton resonance. [Reproduced from ref 79. Copyright 2022, American Chemical Society.] (d) PL spectrum of (3-FPEA)_2_PbI_4_ excited with a pulsed laser tuned above (2.48 eV) and below (2.34, 2.21, and 2.07 eV) the band gap. The line shape of the spectra is very similar for all excitation energies. [Adapted with permission from ref 83. Copyright 2020, Springer Nature.] (e) PL spectrum (black) and in-phase component of the modulated surface photovoltage signal (red) measured on a single (BA)_2_PbI_4_ crystal. [Reproduced from ref 81. Copyright 2024, American Chemical Society.]

The microscopic origin of these emissive features remains under active debate. A growing body of evidence points to strong coupling between photoexcited carriers and lattice vibrations as a key factor shaping the PL response, but not the only one. In particular, emission is frequently associated with the formation of self-trapped excitons,^77,84,88,89^ as well as with radiative recombination from defect-bound excitonic states.^77,82,83^ Both mechanisms typically produce broad, strongly red-shifted emission bands accompanied by pronounced Stokes shifts. Owing to their similar spectral phenomenology, however, they are difficult to disentangle using purely optical techniques, which has led to diverse interpretations of sub-band gap emission in terms of intrinsic^84^ or extrinsic^80^ self-trapped excitons, large-polaron complexes,^78,79^ and defect-induced recombination.^81–83^ In the following, we critically examine the experimental signatures that have been used to support these different mechanisms.

The polar and soft nature of the perovskite lattice naturally leads to an elastic deformation after the photocreation of carriers.^90,91^ The presence of photoexcited carriers introduces a variation in the length of the bond within lead halide octahedra, which leads to rapid localization of carriers in the polarized lattice.^89,92^ There is currently a general consensus that broadband sub-band gap emission observed in ⟨110⟩-oriented^84,85^ and Br or Cl-based 2D perovskites^93,94^ originates from the recombination of self-trapped excitons. In contrast, in the case of ⟨100⟩-oriented 2D perovskites, the origin of the low-energy emission is currently under debate. In addition to the recombination of self-trapped excitons, there is an increasing number of studies that relate low-energy broad-band emission peaks to the presence of defect states.^77,80–83,95,96^ Examples of low energy peaks are visible in the PL spectrum of BA_2_MA_*n*–1_Pb_*n*_I_3*n*+1_ (*n* = 2, 3, 4) 2D perovskites shown in Figure 4(a).^77^ Salient features which allow to assign these peaks to self-trapped excitons or defect-bound excitons include the temperature and power dependence of the PL spectrum. The peaks labeled BE1 in Figure 4(a) were assigned to the recombination of defect-bound excitons, due to the presence of intensity saturation at high excitation power. Moreover, the intensity of peak BE1 exhibits a monotonic trend as a function of temperature.^77^ In the case of self-trapped excitons (peaks labeled BE2 in Figure 4(a)), the energy barrier for their trapping and detrapping is not symmetric.^77,93,97^ This implies that the relative intensity of the PL peak associated with the free exciton and that assigned to the self-trapped exciton displays a nonmonotonic trend as a function of the temperature,^77,93,97^ which was observed for peaks BE2.

Self-trapping has also been used to explain the peculiar emission polarization properties of the low-energy emission of 2D perovskites.^98^ Light emission in semiconductors is generally described within the electric dipole approximation. Other contributions, such as magnetic dipole and electric quadrupole transitions, are usually neglected because their transition rate is orders of magnitude smaller than that of the electric dipole transition.^99^ However, this difference can be mitigated in the presence of (possibly temporary) structural distortions, which can alter the crystal symmetry. This would enable faster magnetic dipole transitions, which can be detected experimentally. By performing energy- and momentum-resolved PL spectroscopy, it has been observed that the low energy peak detected by exciting a 2D perovskites was emitted at angles very close to the total internal reflection at the semiconductor-air interface.^98^ Polarization-resolved measurements revealed that this component, which can be detected with a very large numerical aperture objective or from the side of the sample, as in Figure 4(b), is almost fully s-polarized (linearly polarized, with the polarization axis parallel to the semiconductor-air interface).^98^ Theoretical calculations show that s-polarized light emitted at large angles from the normal direction cannot originate from electric dipole transitions, which would also yield p-polarized emission (linearly polarized perpendicularly with respect to the direction of propagation of the light). Only magnetic dipole transitions can give strongly s-polarized PL emitted at large momenta. This was considered to be strong evidence for the magnetic dipolar origin of the low-energy peak in the PL spectrum of 2D perovskites.^98^

Symmetry analysis demonstrates that the 2p exciton state can radiate in the direction perpendicular to the *c*-axis of the perovskite crystal.^87^ However, the 2p Rydberg state has much larger energies than the 1s state.^75^ To solve this apparent contradiction, strong electron–phonon coupling has been invoked, with the possible formation of a parity-broken self-trapped exciton in which the electron and hole are found in a p-like configuration.^87^ The temperature dependence of the low energy PL peak shown in Figure 4(b) was considered compatible with the contribution of self-trapped excitons and was explained in a configuration coordinate model.^87^ The p-like exciton, routinely considered optically inactive in one-photon spectroscopy,^75^ can couple with s-like exciton states in the presence of antisymmetric distortions.^87^

The strong electron–phonon coupling characteristic of 2D perovskites can induce not only the formation of small polaron states that lead to the self-trapping of excitons,^84^ but also the formation of an exciton polaron, which results from quasi-static lattice deformations. This yields a spectral feature energetically close to the free exciton observed in the reflectance spectrum (typical red shifts are up to a few tens of meV). The dynamics of this formation process have been investigated with time-reolved PL measurements, shown in Figure 4(c). The PL spectrum of PEA_2_PbI_4_ in which a hydrogen atom of the phenyl group has been substituted by Br exhibits a red shift, with a time scale of the order of a few to a few tens of ps.^79^ The amount of this red shift shows a positive correlation with the degree of structural distortion of the specific 2D perovskite compound.^79^ This was considered to support the polaronic origin of the PL red shift, because distorted perovskite lattices have shown more pronounced structural changes after photoexcitation. Moreover, the dynamics of the red shift shown in Figure 4(c) is compatible with the temporal scales associated with lattice relaxation.^100^

Luminescence at energies below the band gap can also be related to the presence of in-gap states, which trap photoexcited carriers.^77,80–83,95,96^ The presence of a broadband emission might reflect the presence of a broad energy distribution of the traps and/or a strong coupling to lattice vibrations. Experimentally, the temperature dependence of the PL spectrum can provide useful information about the nature of the low-energy emission. The observation of a monotonic increase of the intensity of the broadband emission with decreasing temperature is considered a signature of defect-related emission.^82,83^ Sub-bandgap excitation can also be used to discriminate between trap-assisted recombination and self-trapped excitons.^77,83^ Self-trapped excitons are transient species, formed after the interaction of photocreated carriers with the polar lattice of the perovskites. As such, they are not expected to yield any spectral features in linear spectroscopy when excitation is performed below the band gap.^77,83^ Conversely, in-gap states can be excited directly with sub-band gap illumination, although with a reduced efficiency, due to their small absorption cross-section. By performing below-band gap excitation at multiple energies, the broadband emission could be consistently observed in a fluorinated PEAPbI_4_ sample, as shown in Figure 4(d).^83^ In these experimental conditions, the intensity of the PL spectrum was shown to display a linear dependence on the excitation power, which allowed to rule out two-photon absorption processes.^83^

Other experimental approaches to distinguish between trap-assisted recombination and self-trapped exciton recombination are based on the consideration that, in the case of a defect-assisted mechanism, the charge carriers that are not subjected to trapping can diffuse in the presence of an external electrical field, thus contributing to electrical currents. In contrast, self-trapped excitons are electrically neutral and are not expected to contribute to currents. A series of experimental techniques which are sensitive to the separation of photogenerated carriers, combined with PL spectroscopy, has allowed to demonstrate that BAPbI_4_ is a p-type semiconductor, with a large concentration of electron traps.^81^ Surface photovoltage spectroscopy is an experimental technique highly sensitive to the concentration of surface trap states. The comparison of the surface photovoltage and PL spectra of BAPbI_4_ shown in Figure 4(e) demonstrates that the weak low energy PL peak at 2.2 eV occurs at the same energy as the main surface photovoltage peak. This demonstrates that surface-related trap states are responsible for the sub-band gap emission observed experimentally.^81^ Moreover, the observation of the surface photovoltage signal at energies as low 1.2 eV suggests the presence of defects with a very broad energy distribution.^81^

In conclusion, we revisited some puzzling aspects of the optical response of 2D lead halide perovskites. We highlighted that, unlike other layered semiconductors, the interpretation of their optical spectra cannot rely solely on excitonic effects. The reflectance and transmission spectra result from an interplay of an exciton manifold with a very large oscillator strength, fine structure splitting and strong electron–phonon coupling. Additionally, the line shape of these spectra is deeply influenced by the coupling of these excitonic complexes with their photonic environment. Simulations based on the transfer matrix method allowed us to capture the salient characteristics of the experimental spectra. A decisive contribution to the line shape comes from the negative permittivity (photonic stopband), which results from the large oscillator strength of the excitons in 2D perovskites. This results in near-unity reflectance within the photonic stop band, analogous to the Reststrahlen effect observed in the infrared spectral region. Such behavior significantly complicates the interpretation of reflection, transmission, and absorption spectra, as the stop band can dominate over and substantially modify spectral features associated with different excitonic transitions in samples thicker than a few tens of nanometers. Consequently, a fundamental understanding of the excitonic properties of these materials would greatly benefit from investigations of high-quality single crystals with minimal thickness.

The emission spectrum is further complicated by the presence of exciton self-trapping in the lattice distortions induced by the presence of photocreated charge carriers and by the trapping of excitons by defects. Importantly, using purely optical methods may not be sufficient to discriminate between these trapping mechanisms, which generally result in broadband emission below the band gap. Experimental methods based on charge transport might represent a complementary and highly informative approach to a complete understanding of the emission spectrum of 2D perovskites.
