# Correction to “Revisiting the Optical Response of Two-Dimensional Perovskites: Beyond Excitons”

**DOI:** 10.1021/acs.nanolett.6c03117

**Published:** 2026-07-27

**Authors:** Alessandro Surrente, Mateusz Dyksik, Paulina Plochocka, Michał Baranowski

In the original article, corrections are needed to the main text:

1. On page 8035, “the dominant excitonic feature is commonly, followed by high energy sidebands separated by approximately 35–40 meV” should be changed to “the dominant excitonic feature is commonly followed by high energy sidebands separated by approximately 35–40 meV”.
2. On page 8039, “incorporating an additional resonance separated by ∼20 eV into the dielectric response” should be changed to “incorporating an additional resonance separated by ∼20 meV into the dielectric response”.
3. On page 8039, “the expected stop-bandwidth” should be changed to “width of the stop band”.

